# Quality of basic emergency obstetric and newborn care (BEmONC) services from patients’ perspective in Adigrat town, Eastern zone of Tigray, Ethiopia. 2017: a cross sectional study

**DOI:** 10.1186/s12884-019-2307-6

**Published:** 2019-05-30

**Authors:** Betell Berhane, Haftom Gebrehiwot, Solomon Weldemariam, Berhane Fisseha, Samson Kahsay, Alem Gebremariam

**Affiliations:** 1 Department of Midwifery, College of Medicine and Health Science, Adigrat University, Adigrat, Ethiopia; 2 Department of Midwifery, College of Health Science, Mekelle University, Mekelle, Ethiopia; 3 Department of Public health, College of Medicine and Health Science, Adigrat University, Adigrat, Ethiopia; 4 Edagahamus Health Center, Eastern Zone of Tigray, Tigray, Ethiopia

**Keywords:** Quality, Basic emergency obstetric and newborn care, Adigrat, Tigray, Ethiopia

## Abstract

**Background:**

Most of the maternal and newborn deaths occur at birth or within 24 h of birth. Provision of quality Basic Emergency Obstetric and Neonatal Care (BEmONC) is very crucial and the current recommended intervention to prevent maternal and newborn morbidity and mortality.

**Methods:**

An institution based cross-sectional study was conducted among mothers receiving at least one of the signal functions of BEmONC services. A total of 398 women were included in the study. The study participants were selected using a systematic random sampling method. Data was collected using structured interviewer-administered Tigrigna version questionnaire. Data were analyzed using SPSS version 20. Multi-variable logistic regression was used to control the effect of confounders.

**Results:**

The perceived quality of BEmONC was 66.7%, which is poor. Clients scored lower quality rates on aspects such as the availability of necessary equipment, lack of clean and functional shower and toilet and administration of anti-pain during delivery and manual vacuum aspiration (MVA). Quality of BEmONC was lower among rural residents (AOR = 0.273, 95% CI: (0.151–0.830). Whereas, Presence of companion (AOR = 2.259; 95% CI: (3.563–13.452) were found with a higher score of quality of BEmONC compared to their counterparts.

**Conclusion:**

The overall perception of quality of BEmONC services received was poor. Residence, ANC follow-up, and presence of companion during labor or delivery were found to have a significant association with the perceived quality of BEmONC services.

**Electronic supplementary material:**

The online version of this article (10.1186/s12884-019-2307-6) contains supplementary material, which is available to authorized users.

## Background

Worldwide, 15% of the expected births result in life-threatening complications during pregnancy, labor, delivery and post-partum period [1]. World Health Organization (WHO) designed and introduced Emergency Obstetric and Newborn Care (EmONC) to reduce maternal and infant mortality [2]. Though remarkable changes have been recorded, maternal and neonatal mortality rates in Ethiopia are among the highest in the World [3] .

A set of seven key obstetric services, or “signal functions,” has been identified as critical to basic emergency obstetric and newborn care (BEmONC): administration of parenteral antibiotics, administration of parenteral anticonvulsant, administration of parenteral uterotonic agents, removal of retained products (MVA), assisted vaginal delivery; manual removal of placenta and resuscitation of the newborn [4] .

As an intervention, the Federal Ministry of Health of Ethiopia is implementing EmONC services. Provision of quality EmONC service is mandatory to achieve the stipulated goals in the sustainable development goals at reducing the maternal and new-born mortality in Ethiopia and worldwide. According to the Ethiopian health care transformation plan, the health system over the last two decades has been focused on improving coverage of essential health service. It is time to pay great attention to the quality and equity of health service at all levels of the system and a lot remains to be done toward improving quality of care at each level of health system [5].

Quality health service is multidirectional; Donabedian’s theory incorporates participants rating in the advent of assessing the quality of health service delivered.

Taking into account the perspective of clients on the maternal and neonatal health care services enables to rate clients’ satisfaction concerning the services received from the healthcare providers [6]. In Ethiopia, there are studies which assessed availability of EmONC services [3, 7–9]. However, there is sub-optimal knowledge of quality BEmONC service from the clients’ perspective and experience [10]. Therefore, this study was conducted to describe the quality of BEmONC services and factors associated with it among mothers receiving these services. This will help to document the quality of EmONC service from the users’ perspective which is important to develop client centered BEmONC guidelines.

## Methods

### Study area and design

The study was conducted in Ganta-Afeshum district, Eastern zone of Tigray which is located around 903 Kilometers to the North of Addis Ababa, the capital city of Ethiopia. It is one of the rural districts of Eastern zone of Tigray. An institution based cross sectional study was conducted among women receiving BEmONC services in 2017.

### Sample size and sampling procedure

A total of 398 women receiving BEmONC services were included in the study. The sample size was computed using a single population proportion formula considering 62% proportion of mothers satisfied with delivery services in a study conducted previously [8] 95% confidence level, 5% margin of error and considering 10% of non-response rate.

There were three public health institutions providing BEmONC services in Adigrat during the data collection period, two health centres and one general hospital. All of these facilities were included in the study. Pre survey assessment was carried out to determine the average daily flow of mothers receiving the services in the hospital and health centers. Accordingly the expected number of attending women in the specified period of data collection, the sample size was proportionally allocated to the health centers and general hospital. Finally, individual study subject were selected from each facility by using systematic sampling techniques.

All women who were discharged after receiving at least one of the signal functions of BEmONC services were included in the study. However, eligible mothers who were referred to other health facilities or unable to respond for the questionnaire were excluded from the study.

### Data collection procedure

Data collection tools were developed in English based on this study’s objectives to be addressed after reviewing relevant literature [11, 9]. The questionnaire was first prepared in the English language then translated to the local language, Tigrigna (also see Additional files 1 & 2). Back translation to English was also done by language experts to check its consistency. Three midwives with bachelors of Science (BSc) data collectors were recruited from Adigrat University as data collectors to fill the tools, and besides, one integrated emergency surgical officer was recruited to supervise the data collectors. Training was given to both the data collectors and the supervisor. The training focused on the objectives of the study, the data collection tool and procedures of the data collection and detailed contents of the tools. Further emphasis was given on the ethical issues of research and smooth and respect full approach with clients. The questionnaire was filled by face to face interaction with the clients after asking their willingness to participate in the study after briefly explaining its objective. Prior to implementation, the questionnaire was pretested and modifications made accordingly. Strict supervision was made by the supervisor and the principal investigator. Completed questionnaires were collected and assessed for consistency and completeness by the supervisor on daily basis.

### Operational definitions

- ➢ Quality- the extent to which health services for populations increased the likelihood of desired health outcomes and are consistent with current professional knowledge.
- ➢ Magnitude of quality with the service: the responses “Strongly agree (very satisfied)” and “Agree (satisfied) ‟ will be classified as agree (satisfied) and responses “strongly disagree (very dissatisfied)”, “disagree (dissatisfied) ‟ and “neutral‟ as disagree (unsatisfied). Neutral responses will be classified as disagree (dissatisfied) considering that they might represent a way of expressing dissatisfaction in a modest way. This is likely because the interview is undertaken within the health facilities and mothers might be reluctant to express their dissatisfaction feeling of the services they received [9].

### Level of quality score in percentage

Good quality- 75% and above.

Poor quality- 74.9% and below [9].

Patient perspective (experience) is feedback from patients on the course of receiving care or treatment, both the objective facts and their subjective views of it. The factual element is useful in comparing what people say they experienced against what an agreed care pathway or quality standard says should happen [12].

### Measurement of quality

#### Donabedian’s framework

The Donabedian’s framework is based on three dimensions of quality; structure, process and outcome. These three are parameters from which inference can be drawn about quality of health care [6]. So, we used this framework to develop the questionnaire.

### Data analysis

Data entry and clearing was done using Epi info. Data was analysed using statistical packages for social sciences version 20. Descriptive data analysis was done to describe the variables under study. Multivariable logistic regression analysis was done to see the independent effect of each variable on the outcome variable. Variables with *p*-value < 0.25 in the bi-variate analysis were included in the multivariable analysis. Multi-colinearity was checked using the variance inflation factor (VIF), and those with VIF greater than 10 were excluded from the model. Result is presented using Adjusted Odds Ratio (AOR) with its 95% Confidence Interval (CI). Significant association was declared at *p* value < 0.05.

### Ethical consideration

Ethical clearance was obtained from Mekelle University College of health sciences institution review board (IRB) with serial No 046/09. Support letter was obtained from the Tigray Regional Health Bureau and Adigrat town health department and respective health institution to collect verbal data before field activities started. Verbal consent was obtained from the study subjects after explaining the study objectives and procedures. For the participants whose age is less than 18 years verbal informed consent was taken from their legal guardians. The participant’s personal identification was not included in the study questionnaire to maintain anonymity. Confidentiality was maintained throughout the study.

## Results

### Socio-demographic characteristics of respondents

A total of 398 mothers fully responded to the interview making 100% response rate. Near three fourth (71.4%) of the participants were from Adigrat general hospital. Majority (59.8%) of the participants completed high school education (7th to 12th grade), Ninety five percent of the participants were currently married. The mean age of the mothers was 27.4 years with standard deviation of (±5.55) years (Table 1).

**Table 1:** Socio-demographic characteristics of respondents in Adigrat, Eastern zone of Tigray, Ethiopia, 2017. (*n* = 398)

Variable	Frequency	Percent (%)
Age of respondents		
15-19yrs	23	5.8
20-24yrs	103	25.9
25-29yrs	139	34.9
30-34yrs	86	21.6
>35yrs	47	11.8
Residence		
Urban	318	79.9
Rural	80	20.1
Religion		
Orthodox	353	88.7
Muslim	35	8.8
Catholic	10	2.5
Ethnicity		
Tigray	382	96
Afar	12	3
Amhara	4	1
Education		
No formal education	35	8.8
1 to 6th	62	15.6
7th to 12th	238	59.8
Certificate/Diploma	42	10.6
Degree and above	21	5.3
Occupation		
Government employed	63	15.8
NGO/Private company employed	6	1.5
Merchant/business	112	28.1
House wife	202	50.8
Student	15	3.8
Marital status		
Married	378	95
Unmarried	20	5
Husband’s education		
No formal education	42	11.1
1 to 6th	40	10.6
7th to 12th	145	38.4
Certificate/Diploma	84	22.2
Degree and above	67	17.7
Husband’s occupation		
Governmental	123	32.5
NGO/Private company	13	3.4
Merchant/business man	155	41
Daily laborer	51	13.5
Un-employed	9	2.4
Farmer	27	7.1
Monthly HH income		
0-1500ETB	76	19.04
1501-3000ETB	189	47.5
3001-4500ETB	75	18.8
4501-6000ETB	51	12.8
> 6000	4	1.1
Unknown/Refusal	3	0.75

### Current obstetric history of respondents

Out of the 398 mothers, 336 (84.4%) mothers had one to four pregnancies and 62 (15.6%) mothers were grand multiparas having 5 to 8 pregnancies. Three hundred seventy-eight mothers had antenatal care (ANC) follow up. Eighty-eight (22.1%) of the mothers were referred from other facilities. Spontaneous Vaginal Delivery (SVD) was the predominant mode of delivery (83.2%). Out of the total births observed, 7 neonatal deaths and 6 still births were recorded (Table 2).

**Table 2:** Current obstetric history of patients receiving BEmONC services in Adigrat town, Eastern zone of Tigray, 2017. (*n* = 398)

Variables	Frequency	%
Gravidity		
Primi	336	84.4
Multi	62	15.6
ANC follow-up		
Yes	378	95.0
No	20	5.0
Desire of current pregnancy		
Wanted	345	86.7
Unwanted	53	13.3
Type of visit		
Direct/ Planned	310	77.9
Referred	88	22.1
Mode of transportation		
Ambulance	288	72.4
Public transportation	102	25.6
By foot	8	2.0
Time waited to receive service		
< 15 min	381	95.7
15-30 min	14	3.5
30 min-1 h	3	0.8
Presence of companion		
Yes	155	38.9
No	243	61.1
Mode of delivery		
SVD	331	83.2
AVD	37	9.3
Abortion	30	7.5
Health outcome of mother after delivery		
Normal	340	85.4
With complication	58	14.6
Birth outcome of the neonate		
Live birth	355	89.2
Neonatal death	7	1.8
Still birth	6	1.5
Health problem on neonate		
No	337	84.7
Yes	25	6.3
Payment		
No	391	98.2
Yes	7	1.8

### Quality of BEmONC services from patients’ perspective

#### Structure

When we see the overall mothers’ perspective of quality in terms of input, 164 (41.2%) mothers scored above 85% or stated it as good quality. Lack of necessary equipment (30.2%) was the major contributing factor for the reported poor quality of (Table 3).

**Table 3:** Input (structural) factors of quality of BEmONC, in Adigrat town, 2017. (*n* = 398)

Variable	Good N (%)	Poor N (%)
Necessary equipment availability	278(69.8)	120(30.2)
Adequate no of health providers	327(82.2)	71(17.8)
Sufficient rooms, beds and space	344(86.4)	54(13.6)
Sanitation	344(86.4)	54(13.6)
Functional and clean shower and toilet	267(67.1)	131(32.9)

#### Process

Mothers perspective of quality in terms of process, 180 (45.2%) of them scored above 75% or stated as good quality. The major contributing factor for the poor quality was failure of health professionals to counsel the clients (12.3%) on how to take care of their newborn baby (Table 4).

**Table 4:** Process factor of quality of BEmONC in Adigrat town, 2017. (*n* = 398)

Variable	Good N (%)	Poor N (%)
Respect and courtesy by the health providers	383 (96.2)	15 (3.8)
The environment where you were laboring was comfortable.	368 (92.5)	30 (7.5)
Active follow up on the progress of labor/abortion.	368 (92.5)	30 (7.5)
Permission before applying any procedures and examination	364 (91.5)	34 (8.5)
explained the labor progress to you by using your local and clear language	351 (88.2)	47 (11.8)
different member of staff have given you similar advice or information	367 (92.9)	31 (7.1)
Health workers spent enough time for examination.	372 (93.5)	26 (6.5)
verbally encouraged praised and reassured	387 (97.2)	11 (2.8)
got enough care and support during the time of labor.	373 (93.7)	25 (6.3)
Confidence and competence of health providers	379 (95.2)	19 (4.8)
Privacy well kept	385 (96.7)	13 (3.3)
Got enough care and support during delivery/abortion	384 (96.7)	13 (3.3)
Availability of health providers	349 (87.7)	49 (12.3)
support from the staff in breast- feeding	325 (91.3)	31 (8.7)
Received counseling on how to take care of your baby	243 (68.2)	113 (31.8)
Your baby received enough care and support.	316 (90.5)	43 (9.5)
Receive adequate anti pain while MVA was performed	10 (33.3)	20 (66.7)

#### Outcome (satisfaction)

In the satisfaction section of the quality, 138 (34.7%) mothers stated as good (satisfied). In this dimension of quality, the overall counseling that were given to patients and involving them in making decision contribute for the poor provision of quality of care (Table 5).

**Table 5:** The output (satisfaction) factors of quality of BEmONC from patients’ perspective, in Adigrat town, 2017. (*n* = 398)

S No	Variable	Good N (%)	Poor N (%)
1	Respect	387 (97.2)	11 (2.8)
2	Professional respect for your privacy	387 (97.2)	11 (2.8)
3	The number of health workers	349 (87.6)	49 (12.4)
4	Health workers competency and confidence	379 (95.2)	19 (4.8)
5	Communication between doctor, nurse and other health staff	364 (91.4)	34 (8.6)
6	Involved you in decision	352 (88.7)	45 (11.3)
7	The overall Counseling that were given in your stay.	347 (87.2)	51 (12.8)
8	Overall care and support, given	374 (93.9)	24 (6.1)
9	Care and support given for your newborn	334 (91.5)	31 (8.5)

### The overall quality of BEmONC services from patients’ perspective

Quality in this study was assessed by combining the three dimensions the input, process and outcome. The quality is classified as good quality if it scored 75% and above. Otherwise it is classified as poor quality. The overall quality of BEmONC services from patients’ perspective conducted in this study was 66.3% with 95% CI (61.6, 71.4), *P*-value 0.04.

### Factors associated with the quality of BEmONC services from patient’s perspective

On multi-variable, women who came from the rural area had lower odds of quality service (AOR = 0.273; 95%CI: 0.15–0.83). On the other side, women who had ANC follow up had higher odds of quality BEmONC service (AOR = 0.004) 95% CI (0.091 (0.011–0 .723). Moreover, those mothers who were accompanied by their relatives during their labor were with 7 times higher odds of good quality BEmONC service (AOR = 6.9; 95% CI: (6.923 (3.563–13.452) compared to their counterparts (Table 6).

**Table 6:** Association of quality of BEmONC services from patient’s perspective in bivariate and multivariate analysis, in Adigrat town eastern zone of Tigray, 2017

Variable	Good	Poor	COR	AOR	CI 95%	*P* value
Residence						
Urban	222	96		1.0		
Rural	42	38	0.47	0.273	0.15–0.83	0.028*
Education						
No formal education	17	18	0.37	0.071	0.08–3.68	0.54
1 to 6th	41	21	0.78	0.334	0.16–4.97	0.91
7th to 12th	162	76	0.85	0.083	0.16–2.66	0.56
Certificate/Diploma	29	13	4.00	2.303	0.39–21.5	0.18
Degree and above	15	6		1.0		
Husband’s education						
No formal education	23	19		1.0		
1st to 6th	23	17	0.47	0.060	0.20–5.36	0.950
7th to 12th	106	39	0.53	1.207	0.10–1.63	0.206
Certificate/Diploma	59	25	1.07	0.683	0.25–1.88	0.478
Degree and above	48	19	0.72	0.912	0.13–2.63	0.209
Husband’s Occupation						
Governmental	86	37		1.0		
NGO	8	5	1.36	0.61	0.20–5.36	0.950
Private(merchant)	110	45	0.94	0.402	0.10–1.63	0.206
Daily laborer	32	19	1.43	1.88	0.25–1.88	0.478
Unemployed	6	3	0.99	0.410	0.13–1.56	0.209
Farmer	17	10	1.17	0.764	0.20–5.36	0.875
Gravidity						
Primi-gravida	228	108	0.91	0.957	0.377–1.141	0.837
Multi-gravida	36	26		1		
ANC Follow-up						
Yes	254	124	2.04	0.004	0.01–0 .72	0.000*
No	10	10		1.0		
Wanted status of pregnancy						
Wanted	241	104	3.02	0.946	0.59–3.80	0.952
Unwanted	23	30		1.0		
Type of visit						
Planned(direct)	216	94	1.91	0.520	0.24–1.08	0.083
Referred	48	40		1.0		
Presence of companion						
Yes	136	19	6.43	2.259	3.56–13.4	0.002*
No	128	115		1.0		
Mode of delivery						
SVD	240	91	4.55	1.692	0.110	0.641
AVD	13	24	0.93	0.632	0.039	0.938
Abortion	11	19		1.0		
Health outcome of the mother						
Normal	241	99	3.704	0.232	1.191–4.998	0.519
With complication	23	35		1.0		
Birth outcome of the neonate						
Live birth	248	107	0.245	1.184	0.02–1.6	0.995
Neonatal death	2	5	0.772	.886	0.35–3.31	0.956
Still birth	3	3		1.0		
Health problem on neonate						
Yes	16	9	4.555	0.019	0.110	0.223
No	235	102	0.936	0.134	0.039	0.083
Any payment for the servicea						
Yes	3	4	0.374	1.433	0.21–11.1	0.571
No	261	130		1.0		
Necessary equipment availability						
Agree	199	65	1.765	1.399	0.70–2.64	0.291
Disagree	85	49		1.0		
Adequate no of health providers						
Agree	35	12	0.956	0.879	0.188–1.067	0.082
Disagree	229	122		1.0		
Sufficient rooms, beds and space						
Agree	204	60	1.898	1.891	0.972–4.157	0.060
Disagree	86	48		1.0		
Functional and clean shower and toilet.						
Agree	65	49	1.649	0.971	0.822 -2.96	0.191
Disagree	199	85		1.0		
Permission before applying any procedures and examination						
Agree	247	17	1.831	1.528	0.545–4.27	0.420
Disagree	119	15		1.0		
verbally encouraged praised and reassured						
Agree	253	10	0.355	0.291	0.041–2.465	0.254
Disagree	132	2		1.0		
Confidence and competence of health providers						
Agree	251	13	3.387	1.587	0.519–5.29	0.266
Disagree	114	20		1.0		
Privacy well kept						
Agree	257	7	1.323	6.911	0.673–12.7	0.082
Disagree	129	5		1.0		
Received counseling on how to take care of your baby						
Agree	170	94	1.464	1.51	0.938–2.33	0.068
Disagree	65	69		1.0		
Receive adequate anti pain while MVA was performed						
Agree	24	240	0.746	1.597	0.812–3.14	0.175
Disagree	18	116		1.0		
Respect and courtesy from the health professionals						
Satisfied	258	6	2.250	1.988	0.267–15.97	0.377
Dissatisfied	130	4		1.0		

The boldface with asterisk [*] entries show the variables that have a significant association with the quality of BEmONC services from patients perspective in Adigrat town, Eastern zone of Tigray, Ethiopia

## Discussion

The overall magnitude of good quality from patients’ perspective was 66.7% with 95% CI (61.6, 71.4).This result was comparable with the study conducted in Northern region of Ethiopia on the perceived quality of delivery and newborn care services which was 65.62% [11].

Providing quality service is not optional, it is a must to decrease the complications and mortality of the mother as well as their newborn babies. However, a significant number of women rated the service as poor. Lack of the necessary equipment and the quality of counseling on caring for the newborn baby was among the major components poorly addressed in the BEmONC service. Clients could not rely on or be satisfied with a health institution which could not fulfill equipment necessary for the services. After delivery, mother’s attention and care is for her newborn and most of the time women who do not have the experience depend on health providers to give them information on how to take of their newborns. But failure to give this information will have a negative effect for the mothers’ rating on the quality of service. On the contrary, patients experienced higher quality on how the health providers verbally encouraged them during labor pain.

Rural residents were at lower odds of perceived quality of BEmONC service. This could be explained by the difference in the level of expectations between the urban and rural residents. Residence has significant association with the quality in this study. This shows that women who live in urban residence have 53 times higher odds of receiving good quality service compared with those coming from rural residence; this result could be due to decreased level of expectation than those who live urban.

ANC follow up had significant association with the quality of the services (*p* ≤ 0.001) showing that women who did not have ANC follow-up score the quality 96 times higher than those who had the follow-up. This response could be because mothers who had the follow-up are aware of the care that is given during delivery because they are counseled during the follow-up, so they tend to score the quality higher. Similarly a study conducted in rural Tanzania shows there is a significance association with the perceived quality of care (*p* = 0.004) [13].

Women who were accompanied by their relatives were with 7 times higher odds of receiving good perceived quality service. This was also documented in other study where women who had continuous support from their relatives during labor and delivery were more likely to be satisfied than women who did not have support [14, 15].

### Limitation of the study

- ➢ Using only one method to assess the quality of the service, that is based on the response of the clients alone which may be affected by the social desirability bias and interviewers bias.
- ➢ The study being in the health institution might give response favoring the care providers.
- ➢ The cross sectional nature of the study makes difficult to establish the cause and effect relationship between the perceived quality and explanatory variables.

## Conclusion

This study revealed that the overall quality of BEmONC services from patients’ perspective was poor. Clients scored lower quality rates on aspects such as availability of necessary equipment, lack of clean and functional shower and toilet and administration of anti-pain during labor and MVA are some of the factors.

Though it is in line with the available literatures, significant number of the women rated the quality of service poor indicating mismatch between the participants’ expectation and service delivered by the providers. Rating the service as poor was higher among rural resident women. On the contrary, good quality rating was higher among those women who had ANC and were accompanied by their relatives during their labor. Lower rate of quality was reported on the availability of equipment, and client provider communication.

## Additional files

Additional file 1: English questionnaire. This data contains a brief description of the study and information for the study participants and legal guardians. (DOCX 25 kb)

Additional file 2: Tigrigna questionnaire, The English version questionnaire was later translated to the local language Tigrigna. (DOCX 31 kb)

## Abbreviations

AOR: Adjusted odds ratio
BEmONC: Basic Emergency Obstetric and Newborn Care
COR: Crude odds ratio
EDHS: Ethiopia Demographic Health Survey
EMOC: Emergency Obstetric Care
HSDP: Health System Development Plan
HSTP: Health System Transformation Plan
MDG: Millennium Development Goals
MMR: Maternal Mortality Ratio
QOC: Quality of care
UNFPA: United Nations Population Fund
UNICEF: United Nations Children’s Fund
WHO: World Health Organization

## Authors’ information

BB (MSc. In Midwifery), Lecturer in Adigrat University, SW (MSc. In Maternity and RH), HG (MSc in Maternity and RH) PhD candidate at Mekelle University, BF (MPH in Epidemiology and Biostat), SK (MSc in IESO), AG (MPH in Epidemiology), PhD candidate at Addis Ababa University
