# Supplementary material for: Quality of basic emergency obstetric and newborn care (BEmONC) services from patients’ perspective in Adigrat town, Eastern zone of Tigray, Ethiopia. 2017: a cross sectional study
Source: BMC Pregnancy Childbirth. 2019 May 30;19:190. doi: 10.1186/s12884-019-2307-6 (PMC6543605; doi:10.1186/s12884-019-2307-6)
Supplement: Supplementary file 1 — English questionnaire. This data contains a brief description of the study and information for the study participants and legal guardians. (DOCX 25 kb) [file 12884_2019_2307_MOESM1_ESM.docx]

### English Questionnaire

### I. Socio-demographic factors

| **No** | **Questions & filter** | **Coding category** | **Skip To** |
| --- | --- | --- | --- |
| 1.01 | How old are you? | --------------------------Yrs. |  |
| 1.02 | Residence | 1. Urban 2. Rural |  |
| 1.03 | What is your Religion? | 1. Orthodox 2. Muslim 3. Catholic 4. Protestant 5. Other (specify……) |  |
| 1.04 | What is your ethnicity? | 1. Tigray 2. Amhara 3. Oromo 4. Erob 5. Afar 6. Other specify…… |  |
| 1.05 | What is your educational level? | 1. No formal education 2. 1-6^th^   1. 7^th^-12^th^ 2. Certificate 3. Diploma 4. Degree & above |  |
| 1.06 | What is your occupation? | 1. Governmental 2. NGO(private company) 3. Private 4. Unemployed 5. Student 6. Other (specify….) |  |
| 1.07 | What is your marital status? | 1. Single 2. Married 3. Divorced 4. Widowed | If 1, 3, or 4 skip  to 1.10 |
| 1.08 | What is your husband’s education level? | 1. No formal education 2. 1-6^th^   1. 7^th^-12^th^ 2. Certificate 3. Diploma 4. Degree & above |  |
| 1.09 | What is your husband’s occupation? | 1. Governmental 2. NGO 3. Private(Merchant) 4. Daily laborer 5. Unemployed 6. Driver 7. Other (specify………….. |  |
| 1.10 | Average monthly household income in Ethiopian Birr. | ETB |  |

| **Obstetric history** | | | |
| --- | --- | --- | --- |
| 1.11 | Gravidity |  |  |
| 1.12 | ANC follow up | 1. Yes 2. No |  |
| 1.13 | Wanted status of current  Pregnancy | 1. Wanted 2. Unwanted |  |
| 1.14 | Type of visit | 1. Planned(direct) 2. Referred |  |
| 1.15 | Mode of transportation | 1. Ambulance 2. Public transportation 3. Other specify………. |  |
| 1.16 | How long did you wait in this hospital before receiving care from health providers for this last delivery? | 1. <15 minute 2. 15-30 minute 3. 30-1 hour 4. > 1 hour |  |
| 1.17 | Presence of companion  during stay | 1. Yes 2. No |  |
| 1.18 | Mode of Delivery | 1. SVD 2. Assisted vaginal delivery 3. Abortion( spontaneous) |  |
| 1.19 | How was your health condition after giving birth/Abortion? | 1. Normal 2. With complication |  |
| 1.20 | Birth outcome after delivery | 1. Alive 2. Neonatal death 3. Still birth 4. Abortion | If 2, 3 or 4 skip  to 1.21 |
| 1.21 | Was there any health problem on your newborn baby? | 1. Yes 2. No |  |
| 1.22 | Have you had to pay for any services or products during your stay? | 1. Yes 2. No |  |

### Questions on Quality

| NO | Question & Filter | Strongly  disagree | Disagree | Neutral | Agree | Strongly  Agree |
| --- | --- | --- | --- | --- | --- | --- |
|  |  | 1 | 2 | 3 | 4 | 5 |
| 2.01 | Received proper respect and courtesy by the health providers during examination. |  |  |  |  |  |
| 2.02 | The environment where you were laboring was comfortable. |  |  |  |  |  |
| 2.03 | Health workers examined thoroughly and made active follow up on the progress of labor. |  |  |  |  |  |
| 2.04 | Health providers asked permission before applying any procedures and  Examination |  |  |  |  |  |
| 2.05 | Health worker explained the labor progress to you by using your local and clear language. |  |  |  |  |  |
| 2.06 | Have you felt safe because different member of staff have given you similar advice or information about your condition. |  |  |  |  |  |
| 2.07 | Health workers spent enough time for examination. |  |  |  |  |  |
| 2.08 | Health workers verbally encouraged praised and reassured during the time of labor. |  |  |  |  |  |
| 2.09 | You got enough care and support during the time of labor. |  |  |  |  |  |
| 2.10 | It felt that the delivery/Abortion room has every material needed to provide good maternal and newborn care. |  |  |  |  |  |
| 2.11 | It appeared that the health providers look like competent and was confident on their work. |  |  |  |  |  |
| 2.12 | Felt that your privacy was kept in delivery/Abortion room. |  |  |  |  |  |
| 2.13 | You get enough care and support during the time of delivery/Abortion care. |  |  |  |  |  |
| 2.14 | Felt that there was enough number of health providers in the ward. |  |  |  |  |  |
| 2.15 | The health workers were available whenever you needed help. |  |  |  |  |  |
| 2.16 | Felt that there were sufficient rooms, beds and space for laboring and delivering mothers. |  |  |  |  |  |
| 2.17 | The wards kept an acceptable level of sanitation. |  |  |  |  |  |

| 2.18 | Did the labor and delivery ward have functional and clean toilet and shower room? |  |  |  |  |  |
| --- | --- | --- | --- | --- | --- | --- |
| 2.19 | Received enough support from the staff in breast- feeding your baby immediately after birth. |  |  |  |  |  |
| 2.20 | Received counseling on how  to take care of your baby |  |  |  |  |  |
| 2.21 | Your baby received enough care and support. |  |  |  |  |  |
| 2.22 | Receive adequate anti pain while MVA was performed? |  |  |  |  |  |

### III Satisfaction

| No | Questions | Level of satisfaction | | | | |
| --- | --- | --- | --- | --- | --- | --- |
|  |  | Strongly  dissatisfied | dissatisfied | neutral | satisfied | Strongly  Satisfied |
|  |  | 1 | 2 | 3 | 4 | 5 |
| 3.1 | The way staffs have treated you with respect and respected your personal wishes, culture, and religion. |  |  |  |  |  |
| 3.2 | Health professional respect for your privacy during your stay. |  |  |  |  |  |
| 3.3 | By the number of health worker in the labor and delivery, ward. |  |  |  |  |  |
| 3.4 | By health workers competency and their confidence on their job. |  |  |  |  |  |
| 3.5 | The communication between doctor, nurse and other health staff about your treatment and condition. |  |  |  |  |  |
| 3.6 | The way staff involved you in decision about you and your baby condition. |  |  |  |  |  |
| 3.7 | By the overall Counseling that were given in your hospital stay. |  |  |  |  |  |
| 3.8 | By the overall care and support, given during labor and delivery time. |  |  |  |  |  |
| 3.9 | By the care and support given for your newborn baby. |  |  |  |  |  |
