# Supplementary material for: Quality of basic emergency obstetric and newborn care (BEmONC) services from patients’ perspective in Adigrat town, Eastern zone of Tigray, Ethiopia. 2017: a cross sectional study
Source: BMC Pregnancy Childbirth. 2019 May 30;19:190. doi: 10.1186/s12884-019-2307-6 (PMC6543605; doi:10.1186/s12884-019-2307-6)
Supplement: Supplementary file 2 — Tigrigna questionnaire, The English version questionnaire was later translated to the local language Tigrigna. (DOCX 31 kb) [file 12884_2019_2307_MOESM2_ESM.docx]

ናይ ስምምዕነት ቅጥዒ ኣብ ጥዕና ትካላት ንዝወለዳ ኣዶታት ትግርኛ ቅጥዒ

ዝከበርክን ናይዚ ፅንዓት ተሳተፍቲ ከመይ ሓዲርክን/ ከበይ ዊዕልክን?

ስመይ ------------------------------ ይበሃል፡፡ ካብ መቐለ ዩኒቨርሲቲ ኣብ ፅፈትን ዕግበትን ግልጋሎት ክንክን ጥንስን ወሊድን ኣብ ዝግበር መፅናዕቲ ዕላምኡ ብዛዕባ ናይ ኣዴታት ርኢቶ ኣብ ዝረኽብኦ ግልጋሎት ርኢቶ ንክንወስድ እዩ፡፡ ኣብ መወዳእታ እዚ መፅናዕቲ እቲ ብኣኻትክን ዝተገለፁ ክፍተታት ክሕግዘና ስለዝኽእል ዘለክን ርኢቶን ክትህባና ብትሕትና ንሓትት፡፡ ኣብቲ ትህቦኦ ርኢቶ ስምክን ይኹን ካርድ ቁፅርኽን ከምዘይምላእ ቀዲምና ክንሕብር ንፈቱ፡፡ ኣብቲ ፅንዓት ጊዜ ዘካፍላኒ ማንኛውም ዓይነት መረዳዕታ ሚስጥራውነቱ ዝተሓለወ እዩ፡፡ ክትምልስኦ ዘይደለኽንኦ ሕቶ ናይ ዘይምምላስ መብራህርሂ ምሕታትን፤ ካብቲ ፅንዓት ዓርሰን ናይ ምግላል መሰልክን ዝተሓለወ እዩ፡፡ ኣብቶም ዝተጠቀሱ ነጥብታት ማእኸል ብምግባር ዝሰማምዓ እንድሕር ኮይነን እቲ ቃልመሕትት ንኽምልሳልና ብትሕትና ንሓትት፡፡

እዚ ክቡር ሰዓትክን ሰዊእክን ንትህባና ትክክለኛ ሓበሬታ ኣቀዲምና ከነመስግን ንፈቱ፡፡

ናይ ምላሽ ወሃቢት መለለይ ቁፅሪ-------------------------------------------------------

ኣገልግሎት ዝረኸበትሉ ቦታ ሆስፒታል / ጥዕና ጣብያ------------------------------------

ናይ መረዳእታ ኣካቢ ሽም------------------------------------

ዘመሳኸሮ ሱፐርቫይዘር ሽምን ፊርማን -------------------------- ዕለት---------------------

**ማሕበራዊ ድሕረ ባይታ**

| ቁፅሪ | መሕትት | መማረፂ | መብራህርሂ |
| --- | --- | --- | --- |
| 1.01 | ዕድመ | __________________ |  |
| 1.02 | ኣበይ ትነብሪ | 1. ከተማ 2. ገጠር |  |
| 1.03 | ሃይማኖትኪ እንታይ እዩ? | 1. ኦርቶዶክስ 2. ሙስሊም 3. ካቶሊከ 4. ካሊእ ግለፅ……… |  |
| 1.04 | ብሔርኪ እንታይ እዩ? | 1. ትግራይ 2. ኣምሓራ 3. ኦሮሞ 4. ኢሮብ 5. ዓፋር 6. ካሊእ ግለፅ……… |  |
| 1.05 | ደረጃ ትምህርቲ? | 1. ዘይተመሃረት 2. 1-6^ይ^ 3. 7^th^-12^ይ^ 4. ሰርተፍትኬት 5. ዲፕሎማ 6. ዲግሪን ካብኡ ንላዕልን |  |
| 1.06 | ኣበይ ትሰርሒ? | 1. ናይ መንግስቲ 2. ግብረ ሰናይ ድርጅት 3. ናይ ግሊ 4. ናይ ቤት እመቤት 5. ተመሃሪት 6. ካሊእ ግለፅ……… |  |
| 1.07 | ኩነታት ሓዳር? | 1. ሓዳር ዘይብላ 2. በዓልቲ ሓዳር 3. ዝተፋሐት 4. ዝሞታ | እንድሕር 1,3 ወይ 4 ናብ1.10 |
| 1.08 | ናይ ብዓል ቤትኪ ትምህርቲ ኩነታት | 1. ዘይተመሃረ 2. 1-6ይ 3. 7th-12ይ 4. ሰርተፍትኬት 5. ዲፕሎማ 6. ዲግሪን ካብኡ ንላዕልን |  |
| 1.09 | ብዓል ቤትኪ ኣበይ ይሰርሕ | 1. ናይ መንግስቲ 2. ግብረ ሰናይ ድርጅት 3. ናይ ግሊ 4. ሚኒዋለ 5. ስራሕ ዘይብሉ 6. ሾፌር 7. ካሊእ ግለፅ……… |  |
| 1.10 | ናይ ቤትኩም ኣጠቃላሊ ወርሓዊ እቶት ክንደይ እዩ | __________________ ብር |  |
| **ኩነታት ሕርሲን ጥንስን** | | | |
| 1.11 | ክንደይ ሻዕ ጠኒስኪ | ______________ |  |
| 1.12 | ናይ ቅድመ ወሊድ ክትትል ትገብሪ ዶ ነይርኪ | 1. እወ 2. ኣይፋሉን |  |
| 1.13 | እዚ ጥንሲ ደሊኪ ዲኪ ጠኒስክዮ | 1. እወ 2. ኣይፋሉን |  |
| 1.14 | ናብዚ ጥዕና ትካል ብከመይ መፂእኪ | 1. ብባዕለይ 2. ብሪፈራል |  |
| 1.15 | ናብዚ ጥዕና ትካል ብምንታይ መፂእኪ | 1. ኣምቡላንስ 2. ናይ ህዝቢ መጉዓዓዝያ 3. ካልእ ግለጽ…… |  |
| 1.16 | ናብዚ ጥዕና ትካል ምስ መፃኺ ቅድሚ ግልጋሎት ምርካብኪ ንክንደይ ዝኣክል ግዜ ተፀቢኪ | 1. < 15 ደቒቓ 2. 15-30 ደቒቓ 3. 31 ደቒቓ - 1 ሰዓት 4. >1 ሰዓት |  |
| 1.17 | መልዓሊ ኣቲክዶ ነይሩ | 1. እወ 2. ኣይፋሉን |  |
| 1.18 | ድሕሪ ወሊድ/ ምንጻል ዝነበረ ናይ ኣዶ ኩነታት | 1. ፅቡቅ 2. ማሕለኻታት ነይሩ |  |
| 1.19 | ብከመይ ወሊድኪ | 1. ብማህፀን 2. ብመሳርሒ ተሓጊዘ 3. ምንጻል ጥንሲ | እንድሕር 3 ናብ 1.22 |
| 1.20 | ናይ ናፅላ ኩነታት | 1. ብህይወት ዝተወለደ 2. ሞይቱ ዝተወለደ 3. ምስ ተወለደ ዝሞተ | እንድሕር 2, ናብ 1.22 |
| 1.21 | ቆልዓ ምስ ተወለደ ዝኾነ ዓይነት ናይ ጥዕና ጸገም ኔርዎ ዶ | 1. እወ 2. ኣይፋሉን |  |
| 1.22 | ኣብ ፃኒሕትኪ ዝኸፈልክዮ ክፍሊት ኣሎ ዶ | 1. እወ 2. ኣይፋሉን |  |

**ሕቶታት ኣብ ፅፈት ግልጋሎት**

|  | ሕቶ | ብጣዕሚ ኣይሰማማዕን | ኣይሰማማዕን | ሞንጎኛ | ይሰማማዕ | ብጣዕሚ ይሰማማዕ |
| --- | --- | --- | --- | --- | --- | --- |
|  |  | 1 | 2 | 3 | 4 | 5 |
| 2.01 | በዓል ሞያ ክብርን ስርዓትን ብዝተመልኦ መንገዲ ምርመራ ገይርልኪ |  |  |  |  |  |
| 2.02 | ዝሓረስክሉ/ዝደቀስክሉ ከባቢ ምቹው ነይሩ |  |  |  |  |  |
| 2.03 | ሰብ ሞያ ሙሉእ ምርመራ ብምግባር ንከይዲ ሕርሲ/ሕክምና ንጡፍ ክትትል ገይሮምልኪ |  |  |  |  |  |
| 2.04 | ሰብ ሞያ ቅድሚ ኣካላዊ ምርመራን ሕክምናን ምግባሮም ኣፍቂዶምኺ |  |  |  |  |  |
| 2.05 | ሰብ ሞያ ብዝርደኣኪ ቁንቁዋ ጌሮም ብዛዕባ ከይዲ ሕርሲ/ሕክምናኺ ይገልፁልኪ ነይሮም |  |  |  |  |  |
| 2.06 | ዝተፈላለዩ ሰብ ሞያ እቲ ሆስፒታል ብዝሃቡኒ ሓደ ዓይነት ሓሳብን ምኽርን ኣይተደናገርኩን |  |  |  |  |  |
| 2.07 | ሰብ ሞያ እኹል ዝኾነ ግዜ ሂቦም መርሚሮምኒ |  |  |  |  |  |
| 2.08 | ኣብ ግዜ ሕርሲ/ቃንዛ ሰብ ሞያ ብቓል ኣረጋጊኦምኺን ኣተባቢዖምኺን |  |  |  |  |  |
| 2.09 | ኣብ ግዜ ሕርሲ/ቃንዛ ካብ ሰብ ሞያ እኹል ዝኾነ ሓዝን ክንክንን ረኺብኪ |  |  |  |  |  |
| 2.10 | እቲ ክፍሊ መዋለዲ/ምንፃል ጥንሲ ንኣደን ህፃንን ንምክንኻን ዘድልዩ ኩሎም መሳርሒታት ዘማለአ ይመስለኪ. |  |  |  |  |  |
| 2.11 | አቶም ሰብ ሞያ ኣብ ዝሰርሕዎ ስራሕ ብቑዓትን ዓርሰ እምነት ዘለዎምን ኮይኖም ተሰሚዕኪ |  |  |  |  |  |
| 2,12 | ኣገልግሎት ክረክብ ከለኹ ግላዊ ሚስጥረይ ከምዝተሓለወለይ ይስምዐኒ |  |  |  |  |  |
| 2.13 | ኣብ ግዜ ወሊድ /ምፅራግ ጥንሲ እኹል ደገፍን ክንክንን ረኺብኪ |  |  |  |  |  |
| 2.14 | እኹል ቁፅሪ ሰብ ሞያ ዘሎ ይመስለኪ |  |  |  |  |  |
| 2.15 | ሓገዝ ኣብ ዝደለኽሉ እዋን ኩሉ በዓል ሞያ ረኺብኪ. |  |  |  |  |  |
| 2.16 | እኹል ክፍልታት ፣እኹል በዝሒ ዓራትን እኹል መዋለዲን /መፅረጊን መንቀሳቐሲን ቦታ ኣለዎ |  |  |  |  |  |
| 2.17 | እቶም ክፍልታት ብፅቡቕ ፅሬት ዝተትሓዙ እዮም. |  |  |  |  |  |
| 2.18 | ኣብ መፅንሒን መዋሊዲን/ጥንሲ መፅረጊን ክፍሊ ዝሰርሕን ፅሬቱ ዝተሓለወን ሻወር ሽንት ቤት ኣለዎ |  |  |  |  |  |
| 2.19 | ቆልዓ ምስ ወለድኪ ወድያው ጡብ ክተጥቡቢ ካብ ሰብ ሞያ እኹል ደገፍ ረኺብኪ |  |  |  |  |  |
| 2.20 | ህፃንኪ ከመይ ከም ትከናኸንዮ ካብ ሰብ ሞያ እኹል ሓበሬታ ረኺብኪ |  |  |  |  |  |
| 2.21 | ህፃንኪ እኹል ደገፍን ክንክንን ረኺቡ. |  |  |  |  |  |
| 2.22 | እኹል ፀረ ቃንዛ ተገይርልኪ |  |  |  |  |  |

1. **ዕግበት ተገልገልቲ**

| ቑፅሪ | መሕትት | መጠንዕግበት | | | | |
| --- | --- | --- | --- | --- | --- | --- |
|  |  | ብጣዕሚ ዘዕግብ  5 | ዘዕግብ  4 | ሞንጎኛ  3 | ዘየዕግብ  2 | ብጣዕሚ ዘየዕግብ  1 |
| 3.01 | ኣብ እዋን ምርመራ ስብ ሞያ ንድሌታትኪ ፣ባህልኺን ሃይማኖትኪን ብዝነበሮም ክብሪ |  |  |  |  |  |
| 3.02 | ኣብ ፃኒሕትኪ ዝነበረ ናይ ሰብ ሞያ ንግላዊ ሚስጥራትኪ ምሕላው |  |  |  |  |  |
| 3.03 | ኣብ ክፍሊ ሕርስን ጥንስን /ምንፃል ጥንሲ ብዝነበሩ ቁፅሪ ሰብ ሞያ |  |  |  |  |  |
| 3.04 | ሰብ ሞያ ኣብ ስርሖም ብዝነበሮም ብቕዓትን ዓርሰ እምነትን |  |  |  |  |  |
| 3.05 | ብዛዕባ ኩነታትኪን ሕክምናኺን ዶክተር፣ ነርስን ካልኦት ሰብ ሞያን ብዝገብርዎ ምርድዳእ |  |  |  |  |  |
| 3.06 | ብዝዕባ ኩነታትኪን ኩነታት ወድኺን ዝግበር ውሳነ እቶም ስብ ሞያ ንዓኺ ዝነበሮም ተሳትፎ |  |  |  |  |  |
| 3.07 | ኣብ ፃንሕትኪ ብዝተውሃበኪ ኩለመደዳይ ምኽሪ |  |  |  |  |  |
| 3.08 | ብዝተውሃበኪ ኩለመዳይ ደገፍን ክንክንን |  |  |  |  |  |
| 3.09 | ንውላድኪ ብዝተገበረሉ ኩለመዳይ ደገፍን ክንክንን |  |  |  |  |  |
